# Christian Owoo: guidance in the pandemic

**DOI:** 10.2471/BLT.23.031123

**Published:** 2023-11-01

## Abstract

Christian Owoo talks to Gary Humphreys about the guidance challenges faced during the COVID-19 pandemic and the importance of adapting guidance to local needs.

*Q: The first COVID-19 cases arrived in Ghana on 12 March 2020, several weeks after the initial reports from China. How ready were you to deal with them at the Korle-Bu Hospital?*

A: In some ways we were quite prepared. We’d all been watching developments in China, Europe and North America, and were beginning to get some sense of how the disease worked. We were also able to draw on our previous experience with infectious disease outbreaks, and that gave us a head start on things like case finding, isolation and containment. But in other ways, we were not at all ready. This was particularly true of emergency and critical care capacity, for example, something which was of direct concern to me as head of critical care at Korle-Bu.

*Q: What were the specific challenges you faced?*

A: Under-capacity was one. Despite being Ghana’s biggest hospital and one of the biggest in Africa, with 2000 beds, we only had 24 ICU (intensive care unit) beds and they were scattered across the campus in six separate units. There was also a serious lack of staff with the skills needed to deliver the care I thought was going to be needed. I had worked in critical care response at the New Royal Infirmary of Edinburgh during the UK’s (United Kingdom of Great Britain and Northern Ireland) H1N1 Swine Flu outbreak in 2009, and with COVID-19 I was seeing a lot of similarities in terms of acute respiratory distress syndrome with multi-organ involvement. So, I had a pretty good idea of what was going to be required once cases started to come in. Most worrying of all, no-one seemed to be taking the critical care challenge seriously.

*Q: Why do you think that was?*

A: All the focus was on containment, perhaps due to our previous experiences with infectious disease outbreaks, and the preparations we’d made for a potential outbreak of Ebola in 2014, which fortunately never came. I think there was also a lack of appreciation of how important critical care was going to be, and perhaps a bit of denial about how hard COVID-19 was going to hit us. In the end, I called the medical director of my hospital to express my concerns. This was on March 6th, our Independence Day.

“Being ready to change guidance based on feedback seems to me crucial.”

*Q: Did the medical director listen?*

A: He did, and a couple of hours later I was in a hotel room with a group of young doctors who were working on the country’s first COVID-19 guidelines. I hadn’t even been told this was going on, but I was happy to contribute. They wanted me to review their recommendations for clinical management of severe diseases and critical care. They said it would probably just take a couple of hours. In the end, I took a room and stayed there for three days working on the clinical management guidance, including the critical care aspect. They had drafted something based on the Ebola protocols that had been drawn up in preparation for the anticipated spill-over of cases from Sierra Leone and Liberia in 2014. They were helpful in many ways, notably in terms of case finding and isolation, but there was nothing about respiratory distress or airborne transmission.

*Q: How did you fill the gaps, given the general lack of knowledge about the pathogen at that point?*

A: Luckily, I was able to draw on the protocols that I’d worked on in collaboration with my colleagues during the HIN1 outbreak and, after three days, we had our first national COVID-19 manual. Just a couple of days later, our first cases arrived. I remember the first case vividly. We had set up response capacity that included a call centre, and were simulating calls with scripted scenarios as part of the training. A call came through from an ambulance driver, saying he was transporting a man from the airport who was strongly suspected of having COVID-19 to our designated isolation and treatment centre. We were all looking at our scenarios and scratching our heads, and then it became clear that this was not a drill but a real case. There was a bit of pandemonium then, but, thankfully, we had a doctor who had dealt with Ebola cases and he said he would move the patient from the ambulance to the ward.

*Q: Is it fair to say there was a degree of anxiety about transmission?*

A: A lot. For the first few days we were isolating everyone, including people who did not require oxygen. And we were wearing full Tyvek suits (protective clothing) and doing non-touch assessment of patients using just pulse oximetry.

*Q: Were you getting any guidance from outside agencies such as WHO at that point?*

A: Some doctors from WHO helped with our national case management work, and a couple of them worked with us in the national COVID-19 centre that we eventually set up. But in the first few weeks we were like any other country, picking up information as it became available and incorporating it into our national guidelines. Then more evidence-based guidance did start to come in, including guidance from WHO on how to set up a COVID-19 facility, and by May 2020 we had actually revised our first national guideline based on what was coming from WHO and Africa CDC (Africa Centres for Disease Control and Prevention), and for that matter, from the US CDC (Centers for Disease Control and Prevention in the United States of America). At the same time, we were trying to ramp up capacity to provide emergency and critical care.

*Q: How long did it take you to reach an adequate level?*

A: We never reached a level I would consider adequate. Resources were mostly being directed at prevention and containment. And it wasn’t just that we lacked beds and equipment, we lacked staff with the necessary skills. We just couldn’t train them fast enough. We quickly learned that the best way to optimize the use of the resources available was to focus our efforts on catching cases before they arrived at the door of the emergency department. So, we implemented active case search and contact tracing strategies based on maps generated by the Ghana health service and Ghana field epidemiology and laboratory training programme. They allowed us to identify hundreds of cases, 9 out of 10 of which were asymptomatic or mild. That in turn allowed us to prevent people from progressing to severe disease through aggressive treatment of the more serious cases. This included emergency resuscitation and stabilization, and the early administration of oxygen therapy to those who had a blood oxygen saturation of below 94%.

“The pandemic forced us out of our silos.”

*Q: WHO recommended a threshold of 90%. What prompted you to take a different approach?*

A: An appreciation of conditions on the ground. A threshold of 90% might make sense in a high-income country where a patient can probably get into a facility, get oxygen and be escalated to the ICU within an hour or even less, but in many places in Africa, indeed in Ghana, it can take that same patient hours or even days to get the support he or she needs for critical illness because of the various barriers to access. So, we had extensive discussions about this and reached a consensus regarding the 94% threshold.

*Q: Did that not mean that you just massively increased demand for services?*

A: In a way, yes, but it was for services that we could actually provide. Oxygen access was a challenge, but we could at least train people to administer it effectively. The 90% threshold was adopted by many countries in the region, and when I was invited to join virtually the AFRO (WHO Africa Regional Office) guidelines adaptation group for COVID-19 in May 2020, shortly after suggesting the change, I found my AFRO colleagues to be very open to the adaptation. And I give them credit for that. Being ready to change guidance based on feedback seems to me crucial. It feeds into the impact-oriented nimbleness that WHO has been pushing for in developing the living guidance approach.

*Q: To what extent did living guidance help you as a clinician during the pandemic?*

A: It made a big difference because there was a constant need to update prevention and treatment protocols. That said, there were still times when guidance lagged behind. I would also point out that we adopted living practices for our own COVID-19 guidance, revising it seven times in less than three years, and only printing hard copies twice. Going digital meant that guidance was not only more up-to-date, but more accessible because people could download it to their phones or other devices.

*Q: Some studies have suggested that access to research increased during the pandemic, but less than some people expected. What was your experience?*

A: A lot of databases and libraries that you previously had to pay for to get access, actually opened up to us. We continued to access journals available through Research4Life or the CDC’s *Journal of public health in Africa*, but we were also accessing specialist journals that had previously been hidden behind paywalls. More information was also available through professional associations, and there was also a lot more information shared as a result of increased networking. That was one of the silver linings of the pandemic: I made more contacts in the past three years than I made in the previous 16 and with all kinds of clinicians. The pandemic forced us out of our silos.

*Q: You mention silver linings. Were any clouds darker than you had expected?*

A: I was certainly reminded of how we continue to underestimate the need for emergency and critical care. Of course, this is a longstanding challenge that reflects a failure to prioritize the need to address acute illness by governments, international organizations, and health-care system stakeholders. My hope is that the pandemic has changed attitudes, and perhaps the passing of World Health Assembly Executive Board resolution EB152(3), calling for the integration of emergency, critical and operative care into universal health care provision is an indication of that. This is something we really need to address.

